# Poor Sympathetic Compensation During Active Standing Increases the Risk of Morbidity–Mortality in the Post-Surgery of Patients with Severe Calcific Aortic Stenosis

**DOI:** 10.3390/biology14020146

**Published:** 2025-01-30

**Authors:** Nydia Avila-Vanzzini, Anayanci Santana-Ortiz, Daniela Sánchez-Estrada, Rashidi Springall, Abel Lerma, Héctor Herrera-Bello, Martín Calderón-Juárez, Claudia Lerma

**Affiliations:** 1 Faculty of Health Sciences, Universidad Anahuac Mexico, Huixquilucan 52786, Mexico; abel_lerma@uaeh.edu.mx; 2 Departamento de Consulta Externa, Instituto Nacional de Cardiología Ignacio Chávez, Mexico City 04480, Mexico; annasanntanna@gmail.com (A.S.-O.); danielasanchez800@gmail.com (D.S.-E.); 3 Departamento de Inmunología, Instituto Nacional de Cardiología Ignacio Chávez, Mexico City 04480, Mexico; raspringall@yahoo.com; 4 Area de Psicología, Instituto de Ciencias de la Salud, Universidad Autónoma del Estado de Hidalgo, San Agustín Tlaxiaca 42160, Mexico; 5 Staff Cardiológico, Medimanage Research, Mexico City 14050, Mexico; hherrera@medimanage.com.mx; 6 Faculty of Sciences, Universidad Nacional Autónoma de México, Coyoacan 04510, Mexico; martincj@ciencias.unam.mx

**Keywords:** heart rate variability, aortic stenosis, aortic valve replacement

## Abstract

(1) Background: Although all severe calcific aortic stenosis (SCAS) patients have decreased sympathetic compensation to active standing, it has not been studied in patients who underwent aortic valve replacement (AVR). The objective was to assess the association of the heart rate variability (HRV) response to an active orthostatic challenge before AVR with the risk of complications or death during the AVR postoperative period in patients with SCAS. (2) Methods: This observational study included 49 patients. The cardiac autonomic activity was assessed by HRV analysis during supine position and active standing (five minutes each). (3) Results: Twenty-four patients (48.9%) who presented outcomes (complication or death) had a greater left ventricular (LV) mass and a smaller magnitude of change during active standing in both the mean cardiac period and sympathetic predominance. Poor sympathetic compensation to active standing and LV mass were independently associated with the outcome odds ratio (OR) = 4.8 [(1.06, 21.8), *p* < 0.041] and 1.03 [(1.007, 1.062), *p* < 0.013], respectively. (4) Conclusions: In SCAS patients, poor sympathetic compensation in the face of orthostatic challenge and greater LV mass are associated with complications or death after AVR surgery. This approach offers an opportunity to find new criteria to reduce the surgical risk of these patients.

## Simple Summary

Patients with severe calcific aortic valve stenosis have chronic sympathetic hyperactivity and a diminished increase in sympathetic activation when they change from supine position (resting) to active standing. In this work, we tested whether the change in heart rate variability indices, as a measure of cardiac autonomic modulation, was associated with complications or mortality after the patients had surgery to replace the aortic valve. We found that those who had complications or mortality after the surgery had a smaller change in their heart rate variability indices when standing, consistent with smaller sympathetic activation compared to those without complications or mortality. We discuss the physiological alterations that may lead to poor compensation of the sympathetic nervous system when the body faces the physiological challenge of standing up before surgery and how this could be tested in future studies as a potential predictor of complications or death after surgery.

## 1. Introduction

Severe calcific aortic stenosis (SCAS) is currently the most common valve disease because atherosclerotic processes are involved in its physiopathogenesis, accelerating its development [1,2]. Aortic valve stenosis is characterized by overactivation of the sympathetic system and a decreased autonomic adjustment when faced with an orthostatic challenge [3]. On the other hand, it has been seen that from the early stage of aortic valve sclerosis, the sympathetic activity becomes increasingly predominant as the valve disease progresses toward stenosis [3,4]. This condition has been associated with increased surgical risk during aortic valve replacement (AVR) [5]. Surgery, the main form of correction of this valve disease [6], carries implicit risks of morbidity and mortality [1,7,8].

Heart rate variability (HRV) is an accepted method to measure cardiac autonomic function [9]; it has become a tool for measuring risk and prognosis for several clinical conditions, whether or not associated with the cardiovascular system [10], and it offers a non-invasive measurement method that contributes to the prediction of operative risk in these patients [5]. Orthostatic posture in patients with SCAE represents a hemodynamic challenge. It has been reported that the gradient can decrease up to 40% and that vasodilation of the peripheral vasculature can occur when the patient is orthostatic. This is one of the causes that lead patients to present syncope or sudden death [3,11], so it is unclear if this lack of sympathetic compensation that some patients show is associated with higher surgical risk. Therefore, the objective of this study was to assess the association of the HRV response to an active orthostatic challenge before AVR surgery with the risk of complications or death during the AVR postoperative period in patients with SCAS. The primary endpoint was to assess the incidence of complications or death from the start of surgery to the first 30 days of hospitalization or hospital discharge. The secondary endpoint was to determine whether other presurgical clinical or echocardiographic variables were associated with death or complications in the same periods.

## 2. Materials and Methods

### 2.1. Study Participants

The study type was classified according to the following criteria: prospective (the study started before the addressed events and outcome), cross-sectional (the studied variables were measured only once), observational (the researchers did not control the study factors), and descriptive (causality cannot be assumed in the association between HRV before surgery and the surgery’s outcomes). Symptomatic patients over 18 years of age with a diagnosis of SCAS close to AVR surgery [6] were consecutively included to assess HRV before AVR surgery. Patients with other major-to-mild valvular heart disease, with left ventricular ejection fraction <50%, significant coronary artery disease (≥50% in any territory), or with the need for any other extra procedures were excluded. Patients with atrial fibrillation and those taking medications related to the autonomic nervous system were also excluded (Supplementary Materials, Table S1). Patients with surgical accidents or changes in the surgical plan in the operating room were eliminated. Patients with a positive COVID event at any time in their lives were not eligible. All patients were at low risk preoperatively according to the Euroscore II scale.

SCAS was defined according to current echocardiography guidelines, considering a trans-valvular velocity ≥4 m/s, a mean gradient of 40 mmHg, and a valve area by continuity method ≤1 cm^2^ [12].

The study outcome was post-surgical complications and mortality. The assessed post-surgical complications were low cardiac output syndrome (LCOS), re-intervention due to major post-surgical bleeding, pneumonia, mediastinitis, acute renal insufficiency, pleural effusion, and hospitalization within 30 days. LCOS was defined when already in the intensive care unit; after correction of electrolyte and blood gas abnormalities and after adjusting the preload to its optimal value, the patient required inotropic medication (dopamine, dobutamine, levosimendan, or epinephrine) to maintain a systolic blood pressure of at least 90 mmHg and cardiac output at 2.2 L/min/m^2^ for 30 min or more.

### 2.2. Heart Rate Variability (HRV)

#### 2.2.1. ECG Acquisition Protocol

The patients signed an informed consent form and were prepared in the pre-surgical area following the hospital guidelines. On the day of admission, they came fasting and stayed in an office free of external noise. A trained researcher placed a Zephyr band (BH3-BTLE Integrator kit-LG, Zephyr Performance System, Medtronic, Annapolis, MD, USA) on the patient’s chest, with which an electrocardiogram record was recorded for 10 min in the supine position and then for the next 10 min during active standing.

#### 2.2.2. Computational Analysis of HRV

**Figure 1:**
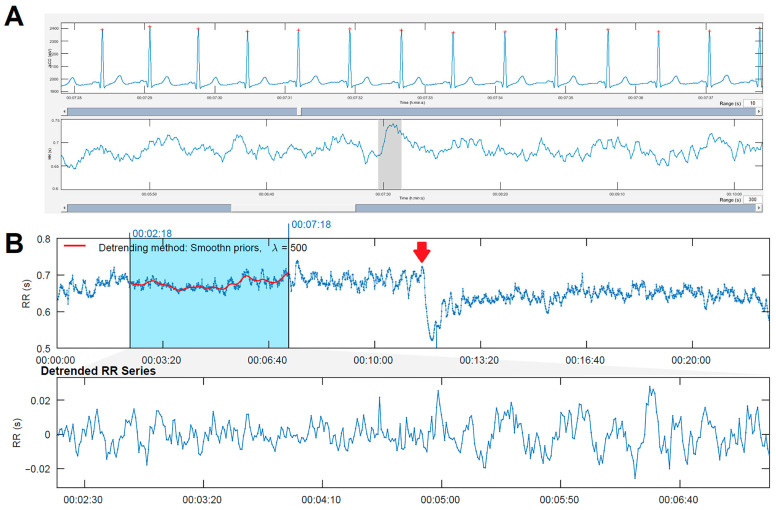
(**A**) Example of electrocardiogram recording with beat identification. Below is the RR interval with the trace where the previous electrocardiogram was taken, indicated by a gray rectangle. (**B**) Recorded RR intervals (over 20 min in length). In the first 10 min (supine position), the heart rate is lower, and the range of RR intervals is between 900 and 1100 ms. Starting at minute 10 (change to active standing), the heart rate is faster: RR intervals range between 700 and 900 ms. The blue rectangle marks the location of the measurement (a 5 min segment), and the red line inside shows the detrending line. Below is the detrended RR time series.

The HRV time series were obtained and analyzed by specialized software (Kubios HRV premium version 4.0) [13]. Figure 1 shows an example of an ECG record obtained from one of the patients, where a small red dot is indicated on top of each R-wave (panel A). The automatic detection of each QRS complex was manually supervised and corrected to obtain the RR intervals (panel B). After identifying the moment of change from supine position to active standing (panel B, red arrow), a five-minute segment was located during supine position (panel B, blue shadow). A filter was applied to substitute any RR intervals involving QRS complexes from premature beats or other arrhythmias. The selected five-minute segments in all patients had no more than 5% of substituted RR intervals [9]. To choose the segment to analyze during the active standing, the first three minutes were left out to achieve better stability.

The RR interval segment of each position was used to analyze the HRV indices described in Table 1. Both time and frequency domain parameters are complementary and correlate with the different components of the autonomous nervous system. For each HRV index, the magnitude of change (Δ) was calculated as the difference between each index in supine position versus active standing.

Furthermore, a dichotomous variable called sympathetic predominance (SP) was constructed, taking into account the reported LF and HF in normalized units (u.n.) as follows: when the percentage of HF > 60 n.u., parasympathetic predominance was considered, and if the LF > 60 n.u., it was considered SP [9].

### 2.3. Statistical Analysis

Categorical variables were expressed in frequency and proportions. The normality of the numerical variables was evaluated with the Kolmogorov–Smirnov test. Depending on the distribution, the results were expressed as mean and standard deviation or median and percentiles (25 and 75). For comparisons, the group was divided according to the presence or absence of the outcome (complication or death). A comparison analysis of means was performed with Student’s *t*-test for quantitative variables with normal distribution. The Mann–Whitney U test was used for quantitative variables with non-parametric distribution and Pearson’s or Fisher’s chi-squared tests were used to compare categorical variables. A univariate and, subsequently, multivariate analysis was performed with binary logistic regression. A two-tailed value of *p* < 0.05 was considered significant. The analysis was performed with the SPSS statistical package (ver. 24.0).

## 3. Results

During the study period, 72 patients met the inclusion criteria, and ten patients were excluded due to a surgical accident (need to repair an iatrogenically affected structure or valve replacement in a case of acute prosthesis dysfunction). On the day of admission, four patients had a positive polymerase chain reaction (PCR) test for COVID-19 and three withdrew their informed consent. In six patients, the immediate post-surgical hemodynamic evaluation was performed with echography and was left to the evaluator’s interpretation. Therefore, it was decided to include only patients who had invasive monitoring (Swan Ganz). In the end, 49 patients were included for analysis, of which 24 [48.9% (95% CI, 35%, 63%)] presented outcomes (complication or death) and 25 patients [51.0% (95% CI, 39%, 66%)] did not have any complications. All patients have a low risk according to the Euroscore II scale. The demographic data of all patients compared by outcome are shown in Table 2. The mean age of the total group was 52 ± 12 years, 30 patients (61%) were men, and there was no statistically significant difference in personal history, pathological or anthropometric variables, or medications. Patients with surgical complications had higher scores on the Euroscore II scale than those without surgical complications.

Table 3 shows the preoperative echocardiogram assessment. There was a statistical difference in left ventricular mass, being higher in the group that presented outcomes (complications or death).

The trans-surgical variables in Table 4 show that this same group had a longer time of mechanical ventilatory support. Twenty-four patients (49%) had complications during and after surgery: LCOS occurred in 14 patients (29%), pneumonia in one patient (4%), mediastinitis in one patient (4%), re-intervention in two patients (8%), acute renal insufficiency in one patient (4%), pleural effusion in one patient (4%), hospitalization after 30 days in three patients (6%), and other complications in three patients (13%). Three patients (13%) died during or after surgery.

Table 5 shows the evaluation of HRV parameters and their relationship with the outcome. The explicit *p*-values of all comparisons are in the Supplementary Materials, Table S2. There was a difference between supine position and active standing in mean RR, RMSSD, and pNN50. Regarding the frequency domain variables, only a difference was observed in LF and HF in the group without complications. When comparing the deltas of change, it was observed that only the mean RR and the sympathetic predominance before active standing were statistically significant, being lower in the group with outcomes (52 ± 12 vs. 110 ± 16 and 4.1 vs. 36%, respectively).

**Figure 2:**
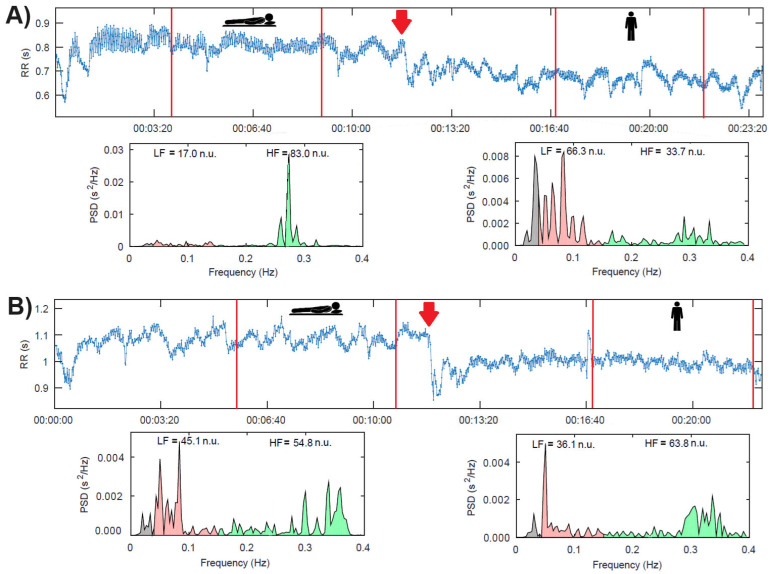
(**A**). Patient with sympathetic predominance in response to active standing. (**B**). Patient without sympathetic predominance in response to active standing. The red arrows indicate the change to an active standing position. The red lines indicate the limits of each selected interval in each position.

Figure 2A shows the example of a patient with a sympathetic predominance (i.e., LF > 60 n.u.) in response to active standing. In contrast, panel B shows a patient with no sympathetic predominance in response to active standing.

In the bivariate with logistic regression (Table 6), mean RR (delta), SP standing, and the left ventricular mass were associated with the outcome (complications or death). Furthermore, the multivariate analysis with one of the variables from the HRV analysis (mean RR delta or SP standing) combined with the left ventricular mass showed that only SP and left ventricular mass were independently associated with the outcome.

**Figure 3:**
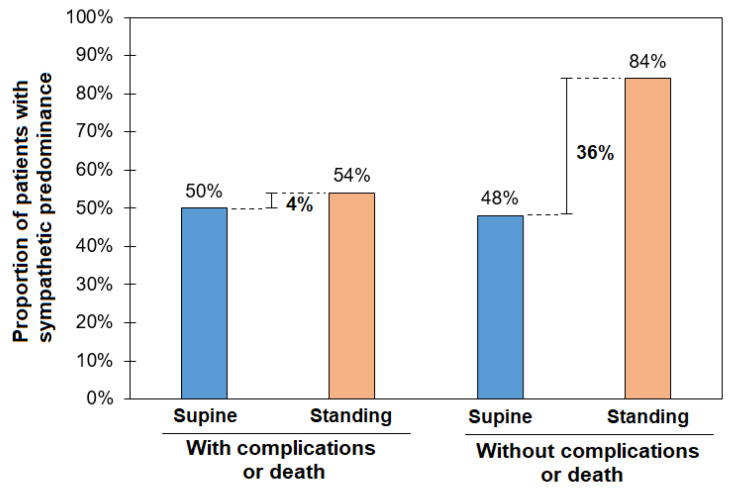
The proportion of patients with a sympathetic predominance (LF > 60 n.u) in the supine position and active standing as a function of the outcome (complications or death).

Figure 3 shows the relationship between dorsal recumbency and orthostatic challenge in patients divided by the presence of the outcome. In patients with the outcome, sympathetic activity does not have a compensation phase with the orthostatic challenge (50% vs. 54%), i.e., they had poor sympathetic compensation with a large proportion of patients without sympathetic predominance while standing. Meanwhile, patients who did not have the outcome showed that they still had a wide range of sympathetic responses to the orthostatic challenge (48% vs. 84%).

## 4. Discussion

The main contribution of this work is to show that patients with SCAS who presented the outcome of complication or death after AVR surgery have poor sympathetic compensation in the face of an active orthostatic challenge in the presurgical phase. The second finding was that greater ventricular mass was associated with a higher incidence of complications or death.

Patients with aortic stenosis show an increase in their sympathetic activity, and this finding increases as the severity of aortic stenosis increases [3,4], as well as its association with the risk of complications or death in AVR surgery [5]. However, despite having a permanent sympathetic overactivity, not all patients can compensate for their hemodynamic state when faced with an orthostatic challenge, and it is precisely this poor sympathetic compensation that was associated with the outcome of death or complication in our study.

In healthy subjects in the supine position, there is a predominance of parasympathetic activity [14,15] manifested with longer RR intervals. In response to active standing, i.e., the orthostatic challenge, the vasomotor centers send an efferent stimulus to the sinoatrial node, and blood pressure and heart rate are adjusted through the sympathetic system and β1 adrenergic receptors. This sympathetic activity manifests itself in the sinoatrial node with a loss of HRV (i.e., RR intervals are shortened), with an increase in the heart rate and with a predominance of LF oscillations [16,17].

In patients with SCAS, it has been reported that they have a downregulation of β1 receptors, and it is thought that this could be related to the degree of heart failure [18,19,20]. The mechanism by which this downregulation of β-adrenergic receptors occurs is not completely elucidated, but an increase in the concentrations of endogenous agonists in the synaptic cleft has been observed, leading to a decrease in receptor density [21,22]. This behavior is similar to what occurs in the scenario of heart failure, and in this last pathology, the administration of β-blockers produces an upregulation of the receptors [23].

The β1 receptors in the myocardium, both in the atria and ventricles, fulfill inotropic and chronotropic functions when the sympathetic system activates them through contact with catecholamines (mainly norepinephrine) [24]. On the other hand, in SCAS, sympathetic overactivity in the supine position and poor sympathetic compensation in orthostatism have been reported in patients with SCAS and contrasted with subjects without aortic valve disease [3]. However, there is no clear answer to whether this scenario of poor compensation for standing puts patients at greater risk when taking them to an AVR. In the study by Zebrowski et al. [5], sympathetic overactivity was associated with the risk of post-surgical morbidity and mortality, which we partially agree with. There are two significant differences between them and our study. First, Zebrowski’s study used a technique based on fractality, whose interpretation in the clinical setting is not completely clear. In contrast, we use the time and frequency domain techniques accepted by the current guidelines for clinical use [9]. Second, we also evaluated patients facing an orthostatic challenge, and we realized that the sympathetic overactivity that our patients have in the supine position is not enough to compensate during the orthostatic challenge in some patients. This point is precisely what is associated with greater risk of complications or death after AVR. We speculate that these patients have less compensation, possibly due to the downregulation of β1 receptors as a manifestation of a stage of heart failure not detected by conventional diagnostic methods such as echocardiography [20,25,26], but that it may be the mechanism of complications following the surgical procedure.

LCOS is one of the most feared complications, with the highest incidence in this study because it predisposes to more significant complications [8,27,28]. Therefore, LCOS and its association with poor sympathetic compensation should be studied. In the present study, a trend was demonstrated between LCOS and poor sympathetic compensation (OR = 2.8 (0.79, 10.5), *p* < 0.108), possibly related to the number of patients included. However, following the same line of ideas, we consider that the poor compensation compared to the orthostatic challenge of the patients who presented the outcome in this study may be related to the low activity of the β-adrenergic receptors and with the poor response in a critical moment such as the immediate AVR postoperative period.

Another unfavorable component of SCAS found in this study was the ventricular mass, with greater hypertrophy in the group with outcomes. This association of significant hypertrophy and complications and/or death has already been reported for several years as a risk factor in cardiac surgery [29] and has to do with the development of pathological hypertrophy in which the presence of fibrosis is implicit [29]. On the other hand, the stimulation of β-1 receptors in the myocardium is a condition that generates an increase in ventricular mass [30]. In the present study, we looked for the association between ventricular mass and increased sympathetic activity without finding it, which can be explained by the collinearity of the variables and the need for a different study design.

Finally, pneumonia and mediastinitis were also post-surgical complications, which, as part of the outcome of the study, could be associated with poor sympathetic response to active standing before surgery. For several decades, multiple animal studies have shown that the function of the immune system is not independent of the central and autonomous nervous system; it is of utmost importance in its proper function, having a clear influence on the acute inflammatory reflex, the resolution of inflammation, and chronic inflammation [31,32]. The prototype reflex circuit that regulates immunity comprises afferent and efferent signals transmitted in the vagus nerve in response to the molecular products of infection and injury, including cytokines and eicosanoids. The activation of adrenergic neurons in the spleen culminates in the release of norepinephrine in the vicinity of T cells capable of secreting acetylcholine. Acetylcholine crosses the marginal zone and enters the red pulp, causing the expression of cytokine-producing macrophages [31]. All these components and many others occur in the normal immune response to effectively regulate inflammation. In observational and experimental studies, it has been found that brain injury, for example, in cases of embolic cerebrovascular events, is related to low immune response and death due to infectious processes such as pneumonia [32]. We consider that in patients with significant aortic valve stenosis in whom autonomic imbalance and poor sympathetic compensation were observed preoperatively, this contributes to the higher incidence of infections, since efficient immune activity requires the balance of both the sympathetic and parasympathetic systems.

### 4.1. Perspectives

Our working group has evidence of an altered evolution of autonomic activity as valvular disease increases [4], with the eventual presentation of decreased sympathetic compensation [3] that is not related to age or other risk factors [33] and of an association between poor compensation of sympathetic activity in the face of orthostatic challenge and a higher risk of death and complications at the time of AVR (current findings). However, the line of research continues, and in the future, there will be evidence of what this poor compensation implies for patients. New studies should be designed in which the downregulation of β-adrenergic receptors is intentionally sought as a predictive mechanism of complications, which may reconsider the optimal timing of AVR surgery. Although the patients in this study had symptoms, no marker of conventional ventricular function was abnormal. It is important to start analyzing patients from a pathophysiological point of view and leave aside the structural and hemodynamic markers currently used to decide on the appropriate surgical moment (trans-valvular velocity or valve area) that are still valid in the guidelines [6]. On the other hand, it is speculated that this poor compensation in SCAS is what predisposes patients to lipothymia, syncope, or even sudden death when faced with common physical challenges [3]. Some studies have emerged that support the drop in cerebral flow in the presence of orthostatic challenge in patients with aortic stenosis, stating that this is a cause of syncope and reflects poor compensatory sympathetic activity [34]. The simple measurement of HRV during the orthostatic test may be useful for monitoring patients with SCAS. With the progress in this line of research, it could become a real tool for determining the best surgical moment for patients and reducing complications and the risk of syncope or sudden death before surgery.

Previous studies on trans-catheter AVR have shown that after 6 months of AVR, the left atrial volume reduction was associated with the improvement in cardiac sympathetic nervous function assessed by 123I-metaiodobenzylguanidine (MIBG) scintigraphy [35]. Further studies with HRV analysis during orthostatic tests before and after surgery could elucidate the contribution of autonomic modulation to the recovery of cardiac function during the first 6 months after AVR.

### 4.2. Study Limitations

This study had a small number of patients. Although differences are observed between groups, these results must be validated with a larger number of participants to increase the statistical precision and to reduce potential type-II errors and with other populations to increase external validity. One of the main limitations to reaching the sample number was the COVID-19 pandemic, which limited patient recruitment. The study included patients with both etiologies of SCAS, namely degenerative disease and bicuspid aortic valve disease, which is reflected in a younger mean age relative to the population affected by degenerative disease. Although the pathophysiology between bicuspid and tricuspid aortic stenosis differs in many aspects, it is similar in others, and even the degenerative inflammatory process is present in bivalve aortic stenosis [36,37]. In our study, the main objective was the consecutive inclusion of patients with severe aortic stenosis near surgery and to evaluate the autonomic system in relation to complications or death independently of the valve’s morphology. Moreover, we took into consideration only the anatomic description that was carried out by the surgeon over the calcified valve with a direct view, and it was found that 21 patients (43%) were carriers of bivalve aorta, which was not associated with the outcome.

To confirm several hypotheses raised in the discussion, new studies are required that include measurements of catecholamines and inflammatory markers, a prospective evaluation where behavior can be monitored and causality confirmed, or a clinical trial in which the reversibility of beta receptor function can be measured through the use of β-blockers, as has occurred in the setting of heart failure.

On the other hand, overactivation of the sympathetic system may be involved in the development of greater ventricular hypertrophy, and this, in turn, is a risk factor for death or complications in cardiac surgery. In the evolution of aortic stenosis and before it reaches a critical state that requires valve replacement, the study of cardiac autonomic modulation in the different phases can help answer the question of whether these patients should be given preventive medications that modulate ventricular remodeling, especially disproportionate ventricular hypertrophy, or it could be a criterion that contributes to the decision of early AVR.

Another limitation of the study was the assessment of blood pressure during the orthostatic challenge by sphygmomanometry, which is known to have low precision. Despite the significant measurement error of this method, we used it to obtain a rough approximation of the blood pressure in order to test differences between groups (with or without surgical complications) and not to test the response of blood pressure to active standing. Further studies with a beat-to-beat measurement of blood pressure are required to evaluate the contribution of SBP and other indices (for instance, the baroreflex sensitivity) in the response to active standing of these patients.

Finally, the volume status of the study participants was not assessed. Since volume status is a predictor of mortality and longer hospitalization after AVR [38], further studies are required to assess the potential influence of plasma volume status on cardiovascular autonomic modulation both before and after AVR and its potential role in mortality and post-surgical complications.

## 5. Conclusions

The study shows that poor sympathetic compensation during the orthostatic challenge in patients with SCAS increases the risk of postoperative complications undergoing AVR. Likewise, greater ventricular mass is a predictor of complications and death. Following this line of research based on HRV analysis, which is inexpensive and has low risk, could be implemented to make earlier decisions regarding the surgical moment for patients and reduce complications.

## Figures and Tables

**Table 1:** Main variables used in the clinical interpretation of HRV [9].

Variable	Description	Autonomic Interpretation
Time domain		
SDNN (ms)	Standard deviation of RR intervals	Sympathetic and parasympathetic
RMSSD (ms)	Square root of the mean of the sum of the squares of differences between adjacent RR intervals	Parasympathetic
pNN50 (%)	Percentage of adjacent RR intervals differing by more than 50 ms	Parasympathetic
Frequency domain		
LF (n.u.)	Mean power in the low-frequency range (0.04 to 0.15 Hz)	Sympathetic
HF (n.u.)	Mean power in the high-frequency range (0.15 to 0.4 Hz)	Parasympathetic and respiratory rate
LF/HF	LF-to-HF ratio	Sympathetic/parasympathetic ratio

n.u. = normalized units.

**Table 2:** Demographic variables of the general group divided according to the outcome.

Variables	All Patients (N = 49)	Group with Surgical Complications (N = 24)	Group Without Surgical Complications (N = 25)	*p*
Age (years)	55 ± 12	57 ± 12	55 ± 14	0.582
Male sex (%)	30 (61)	15 (62)	15 (60)	0.858
Body mass index (kg/m^2^)	27.9 ± 3.8	27.6 ± 4.2	28.4 ± 3.4	0.458
Body surface area (m^2^)	1.76 ± 0.15	1.76 ± 0.17	1.78 ± 0.15	0.700
Body fat (%)	30 ± 10	29 ± 11	32 ± 11	0.315
Muscle mass (%)	31 ± 5	32 ± 6	30 ± 5	0.216
Visceral fat (%)	10 (7, 13)	10 (7, 12)	11 (8, 14)	0.309
Obesity	6 (12%)	2 (8%)	4 (16%)	0.354
Type 2 diabetes mellitus	9 (18%)	5 (21%)	4 (16%)	0.725
Hypertension	22 (44%)	10 (41%)	12 (48%)	0.656
Smoking	17 (34%)	8 (33%)	9 (36%)	0.845
Dyslipidemia	17 (34%)	10 (41%)	7 (28%)	0.315
Hypothyroidism	3 (6%)	1 (4%)	2 (8%)	0.516
Supine position SBP (mmHg)	110 (110, 130)	110 (110, 130)	120 (110, 130)	0.887
Active standing SBP (mmHg)	110 (110, 130)	110 (105, 125)	120 (110, 130)	0.383
ACEI or ARB	21 (43%)	10 (42%)	11 (44%)	0.549
Calcium antagonist	4 (8%)	2 (8%)	2 (8%)	1.000
Statins	19 (39%)	7(29%)	12 (48%)	0.052
Diuretics	12 (24%)	8 (33%)	4 (16%)	0.288
Other	25 (51%)	13 (54%)	12 (48%)	0.310
Euroscore scale	0.64 ± 0.21	0.72 ± 0.27	0.57 ± 0.05	0.011
Bivalve aorta	21 (43%)	10 (42%)	11 (44%)	0.869

SBP = systolic blood pressureACEI = angiotensin-converting enzyme inhibitor; ARB = angiotensin receptor blocker; Other: levothyroxine, metformin, dapagliflozin, insulin.

**Table 3:** Echocardiographic assessment before surgery and morbidity–mortality after surgery. Data are shown as mean ± standard deviation, median (percentile 25, percentile 75), or absolute value (percentage).

Variables		Morbidity–Mortality After Surgery		*p*
	Total (N = 49)	Yes (N = 24)	No (N = 25)	
Diastolic diameter (mm)	44 ± 6	44 ± 7	44 ± 5	0.699
Systolic diameter (mm)	28 ± 6	27 ± 6	28 ± 6	0.388
Left ventricle mass (gr/m^2^)	113 ± 35	129 ± 36	98 ± 27	0.001
End-diastolic volume (mL)	80 (57, 101)	80 (60, 119)	71 (57, 94)	0.555
End-systolic volume (mL)	23 (20, 36)	22 (20, 36)	25 (22, 36)	0.342
Left ventricle ejection fraction (%)	61 ± 6	60 ± 7	62 ± 5	0.210
Diastology				
Grade 1 Grade 2 Grade 3	35 (71%) 12 (24%) 2 (4%)	15 (62%) 7 (29%) 2 (8%)	20 (80%) 5 (20%) 0 (0%)	0.144
Left atrium vol (mL/m^2^)	39 ± 11	40 ± 12	38 ± 11	0.642
E/E′	11 (9, 14)	12 (10, 15)	10 (9, 13)	0.189
Right ventricle base (mm)	36 ± 5	36 ± 5	36 ± 5	0.849
Fractional shortening area (%)	48 ± 8	47 ± 8	49 ± 9	0.582
TASE (mm)	22 (20, 25)	23 (21, 28)	22 (20, 24)	0.340
PSAP (mmHg)	31 ± 9	32 ± 11	30 ± 6	0.447
Vmax (m/s)	5.0 (4.5, 5.5)	5.1 (4.65, 5.8)	4.9 (4.4, 5.2)	0.279
Aortic valve area (cm^2^)	0.6 (0.5, 0.7)	0.6 (0.4, 0.7)	0.6 (0.6, 0.8)	0.110

PSAP = pulmonary systolic arterial pressure; TASE = tricuspid annulus systolic excursion; Vmax = maximum aortic valve velocity.

**Table 4:** Trans-surgical variables and morbidity–mortality after surgery. Data are shown as mean ± standard deviation or median (percentile 25, percentile 75).

		Morbi-Mortality After Surgery		*p*
Variables	Total (N = 49)	Yes (N = 24)	No (N = 25)	
Extracorporeal circulation time (min)	126 (104, 149)	132 (99, 159)	122 (105, 146)	0.638
Aortic clamping time (min)	98 ± 25	101 ± 28	95 ± 22	0.397
Extubation (h)	12 (6, 24)	24 (13, 40)	7 (6, 12)	<0.001

**Table 5:** HRV parameters and their relationship with the outcome.

	Complication or Death (N = 24)			Without Complication or Death (N = 25)		
Variable	Supine Position	Active Standing	Change (Δ)	Supine Position	Active Standing	Change (Δ)
Mean RR (ms)	875 ± 139	823 ± 124 **	52.9 ± 60.9	908 ± 161	798 ± 171 **	110.2 ± 80.7 **^&^**
SDNN (ms)	24.3 ± 15.9	204 ± 10.0	3.9 ± 8.9	29.0 ± 34.0	28.8 ± 46.4	0.2 ± 14.3
RMSSD (ms)	19 (14, 26)	13 (11, 22) **	3.4 (0.9, 7.5)	21 (15, 32)	14 (9, 26) *	3.8 (−0.41, 11.6)
PNN50 (%)	1.0 (0.0, 4.1)	0.3(0.0, 2.0) *	0.50 (0.0, 2.4)	1.0 (0.0, 6.5)	0.0 (0.0, 6.5) *	0.2 (0.0, 3.2)
LF (n.u.)	59 (39, 71)	60 (43, 79)	−4.4 (−18.8, 6.20)	60 (47, 67)	70 (59, 72) *	−6.60 (−28.3, 5.0)
HF (n.u.)	39 (27, 60)	38 (20, 56)	4.2 (−6.1, 18.4)	39 (32, 52)	29 (27, 39) *	6.5 (−5.0, 28.0)
LF/HF ratio	1.51 (0.66, 2.5)	1.58 (0.77, 3.81)	−0.39 (−1.07, 0.24)	1.54 (0.89, 2.0)	2.34 (1.50, 2.68)	−0.87 (−1.65, 0.37)
SP (LF > 60 nu)	12 (50%)	13 (54%)	1 (4.1%)	12 (48%)	21 (84%) **	9 (36%) **^&^**

* *p* < 0.01, ** *p* < 0.001 difference between bodily positions; ^&^ *p* < 0.01 difference between **Δ**; SP = sympathetic predominance.

**Table 6:** Bivariate and multivariate logistic regression analysis of HRV using deltas of change, SP standing, and left ventricular mass (independent variables) and its relationship with complications or death (dependent variable).

	Bivariate Regression		Multivariate Regression Model 1		Multivariate Regression Model 2	
Variable	OR (CI, 95%)	*p*	OR (CI, 95%)	*p*	OR (CI, 95%)	*p*
Mean RR (delta)	0.987 (0.976, 0.997)	0.01			0.99 (0.98, 1.00)	0.061
RMSSD delta	1.03 (0.97, 1.09)	0.30				
pNN50 delta	0.981 (0.919, 1.04)	0.55				
LF delta	1.01 (0.987, 1.04)	0.32				
HF delta	0.987 (0.962, 1.01)	0.32				
LF/HF delta	1.005 (0.783, 1.29)	0.96				
SP standing (LF > 60 nu)	5.25 (1.38, 19.9)	0.02	4.82 (1.066, 21.8)	0.04		
Left ventricular mass (gr/m^2^)	1.03 (1.01, 1.05)	<0.01	1.03 (1.007, 1.06)	0.01	1.03 (1.01, 1.06)	0.02

## Data Availability

The raw data supporting this article’s conclusions will be available upon request to the corresponding author, provided the pertinent legal requirements are met.

## Supplementary Materials

The following supporting information can be downloaded at: https://www.mdpi.com/article/10.3390/biology14020146/s1, Table S1. List of medications that may affect the autonomic nervous system; Table S2. Statistical significance (*p* values) of comparisons of HRV parameters.
