# Supplementary material for: Poor Sympathetic Compensation During Active Standing Increases the Risk of Morbidity–Mortality in the Post-Surgery of Patients with Severe Calcific Aortic Stenosis
Source: Biology (Basel). 2025 Jan 30;14(2):146. doi: 10.3390/biology14020146 (PMC11851686; doi:10.3390/biology14020146)
Supplement: Supplementary file 1 [file biology-14-00146-s001.zip › Supplementary Material.pdf]

Supplementary Material

Table S1. List of medications that may affect the autonomic nervous system.

Medication	Pharmacological action
Epinephrine	The active sympathomimetic hormone from the adrenal medulla. It stimulates alpha- and beta-adrenergic systems, causes systemic vasoconstriction and gastrointestinal relaxation, stimulates the heart, and dilates bronchi and cerebral vessels. It is used in asthma and cardiac failure and to delay absorption of local anesthetics.
Clonidine	An imidazoline sympatholytic agent that stimulates alpha-2 adrenergic receptors and central imidazoline receptors. It is commonly used in the management of hypertension.
Phenylephrine	An alpha-1 adrenergic agonist used as a mydriatic, nasal decongestant, and cardiotonic agent.
Formoterol Fumarate	An adrenergic beta-2 receptor agonist with a prolonged duration of action. It is used to manage asthma and treat chronic obstructive pulmonary disease.
Isoproterenol	Isopropyl analog of epinephrine; beta-sympathomimetic that acts on the heart, bronchi, skeletal muscle, alimentary tract, etc. It is used mainly as a bronchodilator and heart stimulant.
Methyldopa	An alpha-2 adrenergic agonist that has both central and peripheral nervous system effects. Its primary clinical use is as an antihypertensive agent.
Metaproterenol	A beta-2 adrenergic agonist is used in the treatment of asthma and bronchial spasms.
Oxymetazoline	A direct-acting sympathomimetic used as a vasoconstrictor to relieve nasal congestion.
Albuterol	A short-acting beta-2 adrenergic agonist is primarily used as a bronchodilator agent to treat asthma.
Salmeterol Xinafoate	A selective adrenergic beta-2 receptor agonist that functions as a bronchodilator when administered by inhalation. It is used to manage the symptoms of asthma and chronic obstructive pulmonary disease.
Terbutaline	A selective beta-2 adrenergic agonist is used as a bronchodilator and tocolytic.
Pilocarpine	A slowly hydrolyzed muscarinic agonist with no nicotinic effects. Pilocarpine is used as a miotic and in the treatment of glaucoma.
Galantamine	It is a cholinesterase inhibitor that has been used to reverse the muscular effects of gallamine triethiodide and tubocurarine and has been studied as a treatment for Alzheimer's disease and other central nervous system disorders.
Donepezil	An indan and piperidine derivative that acts as a selective and reversible inhibitor of acetylcholinesterase. Donepezil is highly selective for the central nervous system and is used in the management of mild to moderate Alzheimer's disease.
Physostigmine	A cholinesterase inhibitor that is rapidly absorbed through membranes. It can be applied topically to the conjunctiva. It can also cross the blood-brain barrier and is used when central nervous system effects are desired, such as in treating severe anticholinergic toxicity.

Pyridostigmine Bromide	A cholinesterase inhibitor with a slightly longer duration of action than neostigmine. It is used in the treatment of myasthenia gravis and to reverse the actions of muscle relaxants.
Rivastigmine	A carbamate-derived reversible cholinesterase inhibitor that is selective for the central nervous system and is used for the treatment of dementia in Alzheimer’s disease and Parkinson's disease.
Tacrine	A cholinesterase inhibitor that crosses the blood-brain barrier. Tacrine has been used to counter the effects of muscle relaxants, as a respiratory stimulant, and in the treatment of Alzheimer’s disease and other central nervous system disorders.
Benztropine	A centrally active muscarinic antagonist that has been used in the symptomatic treatment of Parkinson's disease. Benztropine also inhibits the uptake of dopamine.
Trihexyphenidyl	One of the centrally acting muscarinic antagonists used for the treatment of Parkinsonian disorders and drug-induced extrapyramidal movement disorders and as an antispasmodic.
Dicyclomine	A muscarinic antagonist is used as an antispasmodic and in urinary incontinence. It has little effect on glandular secretion or the cardiovascular system. It does have some local anesthetic properties and is used in gastrointestinal, biliary, and urinary tract spasms.
Scopolamine	Scopolamine and its quaternary derivatives act as antimuscarinics like atropine but may have more central nervous system effects. Its many uses include an anesthetic premedication, treating urinary incontinence and motion sickness, an antispasmodic, and a mydriatic and cycloplegic.
Propantheline	A muscarinic antagonist is used as an antispasmodic in rhinitis, in urinary incontinence, and in the treatment of ulcers. At high doses, it has nicotinic effects that result in neuromuscular blocking.
Ipratropium	A muscarinic antagonist structurally related to atropine but often considered safer and more effective for inhalation use. It is used for various bronchial disorders, in rhinitis, and as an antiarrhythmic.
Tiotropium Bromide	A scopolamine derivative and cholinergic antagonist that functions as a bronchodilator agent. It is used in the treatment of chronic obstructive pulmonary disease.
Oxybutynin	Oxybutynin has both a direct antispasmodic effect on bladder detrusor smooth muscle and an anticholinergic effect by blocking the muscarinic effects of acetylcholine on smooth muscle.
Adrenergic beta-Antagonists	Drugs that bind to but do not activate beta-adrenergic receptors thereby blocking the actions of beta-adrenergic agonists. Adrenergic beta-antagonists are used for treatment of hypertension, cardiac arrhythmias, angina pectoris, glaucoma, migraine headaches, and anxiety.

Table S2. Statistical significance (***P*** values) of comparisons in HRV parameters.

	Supine position vs active standing (within the same group)		Group with complications or death vs group without complications or death		
	**Complications or death**	**Without complications or death**	**Supine position**	**Active standing**	**Change (Δ)**
Mean RR (ms)	0.001	< 0.001	0.453	0.566	0.007
SDNN (ms)	0.927	0.115	0.538	0.387	0.284
RMSSD (ms)	0.001	0.011	0.719	0.424	0.952
PNN50 (%)	0.010	0.039	0.655	0.533	0.920
LF (n.u.)	0.189	0.035	0.968	0.401	0.435
HF (n.u.)	0.219	0.037	0.920	0.390	0.447
LF/HF ratio	0.092	0.088	0.952	0.401	0.646
SP (LF > 60nu)	1.000	0.057	0.558	0.012	0.012
